# Kinetics, isotherms, and thermodynamics study on the dissolution-assisted conversion of plastic and biomass wastes into activated carbon

**DOI:** 10.1039/d5ra07621h

**Published:** 2026-02-12

**Authors:** Junaid Saleem, Zubair Khalid Baig Moghal, Gordon McKay

**Affiliations:** a Division of Sustainable Development, College of Science and Engineering, Hamad Bin Khalifa University, Qatar Foundation Doha Qatar jsaleem@hbku.edu.qa zubairkhalid009@gmail.com

## Abstract

Plastic and biomass wastes present persistent environmental challenges due to low recycling efficiency and limited value recovery. Polyolefin waste is particularly difficult to process, whereas palm fronds (PF) are an abundant lignocellulosic resource. This study focuses on the kinetics, isotherms, and thermodynamics of cationic dye adsorption using activated carbon (AC) derived from these wastes *via* a dissolution-assisted conversion route combining chemical activation and surface functionalization. The ACs were characterized for their morphology, surface area, pore structure, and surface chemistry, and their adsorption performance was evaluated. Equilibrium data were analyzed using six isotherm models, with the Langmuir model showing the best fit, reflecting monolayer adsorption with maximum capacities of 841 mg g^−1^ and 707 mg g^−1^ for Rhodamine B and Nile Blue, respectively. Kinetic studies revealed rapid initial uptake reaching equilibrium within 6 h, while thermodynamic evaluation confirmed a spontaneous and endothermic adsorption process. Overall, this work offers a sustainable and scalable route to convert mixed wastes into efficient adsorbents for dye-laden wastewater treatment.

## Abstract 2

Plastic and biomass wastes present persistent environmental challenges due to low recycling efficiency and limited value recovery.

## Graphical abstract

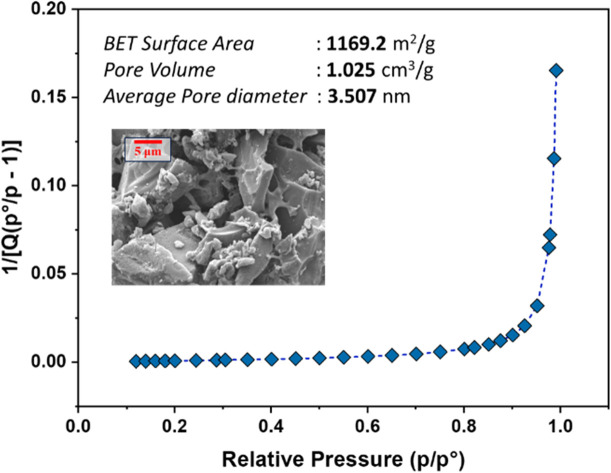

## 1. Introduction

Mixed plastic waste (MPW) continues to impose serious environmental and economic challenges owing to its durability, poor recycling efficiency, and unsustainable end-of-life disposal practices. Globally, less than 10% of plastics are recycled.^1,2^ A 2017 analysis reported that plastic packaging loses about 95% of its material value after a single use, leading to an estimated annual economic loss of USD 120 billion.^3^ These statistics underscore the urgent need for innovative valorization routes that can simultaneously reduce environmental damage and recover economic value.

Polyolefins, predominantly polyethylene (PE) and polypropylene (PP), account for nearly 60% of total MPW, making them the most problematic fraction in plastic waste management.^4,5^ In 2022, PE and PP production reached 110 and 79 million metric tons, and by 2030, these figures are expected to surpass 135 and 105 million metric tons, reflecting the continued growth in polyolefin demand worldwide.^6,7^ This continued expansion magnifies the ecological burden associated with polyolefin waste and emphasizes the need for advanced recycling and conversion technologies.

In parallel, biomass-derived activated carbon (AC) has attracted significant interest as a sustainable material that merges waste utilization with high-performance environmental applications.^8,9^ Such ACs offer the benefits of low energy demand, reduced carbon footprint, and superior textural properties—including high surface area, porosity, and electrical conductivity—which make them highly effective for adsorbing dyes and organic pollutants from wastewater.^10,11^

A particularly promising direction involves integrating plastic and biomass wastes into a unified synthesis pathway for AC production.^12,13^ Biomass precursors offer renewable, low-cost, and widely available feedstocks for the preparation of AC, supporting circular economy objectives. Studies have demonstrated the potential of a broad range of agricultural residues for AC preparation,^14,15^ such as pomace leaves,^16–18^ date pits,^19–22^ olive stones,^23–25^ palm fiber,^26,27^ palm fronds (PF),^28,29^ , bamboo,^30^ coconut shells,^31,32^ and *Azolla filiculoides*.^33^ Among these, PF represents a highly abundant lignocellulosic feedstock capable of yielding high-quality AC upon pyrolysis and chemical activation. However, conventional powdered AC suffers from poor recovery in aqueous systems.

Accordingly, attention has turned to structured forms of AC, including granular AC,^36^ composites and membranes,^34^ membranes and composites,^35,36^ carbon monoliths,^37–39^ and flakes^40^—to enhance recovery, regeneration, and reusability. These structured configurations improve process sustainability and long-term operational efficiency in water treatment systems.

However, many of these fabrication strategies rely on expensive precursors, complex synthesis routes, or binders that compromise adsorption efficiency. The use of inert binders and the inherently larger particle size of granular AC often reduce accessible surface area and active adsorption sites, resulting in lower adsorption capacities compared to powdered AC. To overcome these challenges, this study develops a dissolution-assisted co-processing route that incorporates thermoplastic polyolefins (PE and PP) during AC synthesis. Unlike conventional binders that serve only as structural agents, the polyolefin phase actively contributes to pore development, generating a highly porous and interconnected structure upon dissolution and carbonization. The resulting flake-like AC composites combine the adsorptive efficiency of powdered AC with the mechanical stability and recoverability of structured forms, while simultaneously offering a sustainable valorization route for low-recyclability polyolefin wastes.

Building on the previously reported life cycle assessment (LCA) of this synthesis route,^41,42^ the present work extends the investigation to the adsorption behavior and mechanistic understanding of these hybrid materials. Through comprehensive kinetic, isotherm, and thermodynamic analyses, the study elucidates adsorption rates, thereby establishing the fundamental principles governing dye removal performance. The combined use of PF activation and dissolution-assisted processing of polyolefin waste provides a practical approach that is both environmentally sustainable and effective for understanding and improving dye adsorption.

## 2. Materials and methods

PF were cut (1–2 cm), oven-dried, and chemically activated using 2 M KOH or NaOH solutions. The soaked samples were washed, re-dried, and pyrolyzed at 550 °C for 3 h under N_2_ (20 °C min^−1^) to produce AC. For composite preparation, semi-crystalline polymers (2 g; PE:PP:UHMWPE = 1:1:2) were dissolved in 50 mL xylene at 130 °C till a homogenous mixture is formed. Then AC (48 g) was added to the solution and allowed to stir till homogeneity is obtained. Then the hot solution was casted into desirable molds, upon cooling the polymer solidifies and solvent is separated. To extract the solvent completely, vacuum condensation is applied resulting in a fragile mold-shaped composite. This composite was subjected to annealing in hot air oven at 170 °C for 20 minutes or up to the melting point of polymer, such that the polymer melts and forms a strong network by enhancing intermolecular interactions. The resulting composite can be cut into desired shapes such as flakes, cubes, or other geometries.

## 3. Results and discussion

For the removal of cationic dyes such as RhB and NB, base activation was employed, as acid activation is typically more suitable for anionic dye removal. Preliminary experiments were conducted using both NaOH and KOH as activating agents, and based on the comparative results (Table 1), KOH activation was selected for all subsequent experiments due to its superior performance. In the preparation of the polymer–carbon composite, xylene was deliberately chosen as the solvent because it effectively dissolves both PE and PP without affecting the carbon matrix. In comparison, toluene dissolves low-density PE but not PP, while naturally derived solvents such as limonene and cymene have limited availability and require higher energy for removal due to their elevated boiling points. Xylene, with its low boiling point and ready availability, can be efficiently removed under mild vacuum conditions, minimizing residual solvent in the final composite.

**Table 1:** Adsorption for NaOH *vs.* KOH activation routes

Temperature (*K*)	Dye	Adsorption (mg g^−1^)	
		KOH	NaOH
298	Rhodamine B	841	712
298	Nile blue	707	548

### 3.1. Textural and morphological characteristics

**Fig. 1:**
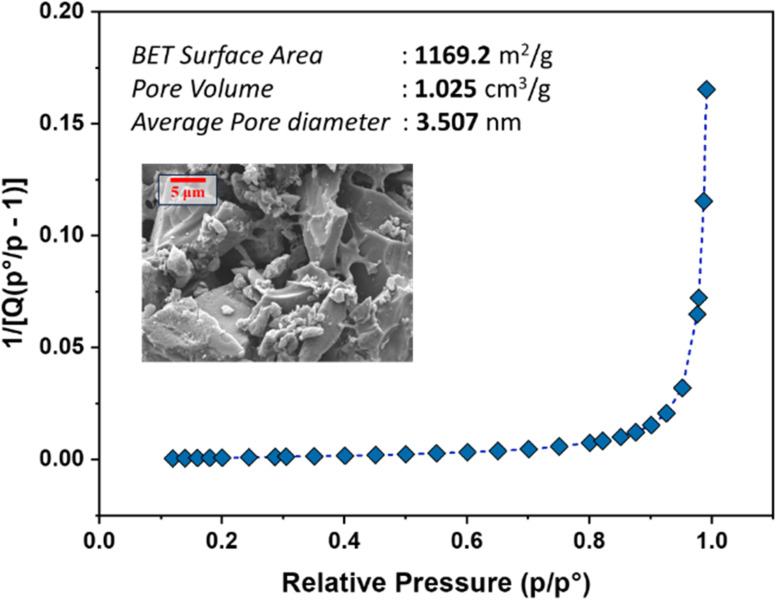
BET surface area plot with an SEM inset.

BET analysis revealed that the AC possesses a high surface area of 1169 m^2^ g^−1^, a pore volume of 1.025 cm^3^ g^−1^, and an average pore size of 3.5 nm, indicating a predominantly micro- and mesoporous structure (Fig. 1). Potassium incorporation enhanced the pore volume, contributing to the substantial surface area.^43^ These textural properties are essential for efficient adsorption by providing ample sites for adsorbate capture, in agreement with previous studies.^44,45^

SEM imaging of the AC (inset of Fig. 1) reveals a porous network with textured carbon regions and embedded polymeric domains. This microstructure confirms the composite nature of the material, combining carbon and residual polymer structures. The combination of high surface area, suitable pore size, and porous network supports effective diffusion and adsorption,^25^ consistent with enhanced pollutant removal observed in similar polymer- or acid-activated biomass-based adsorbents.^46^

### 3.2. Structural and surface chemistry analysis

**Fig. 2:**
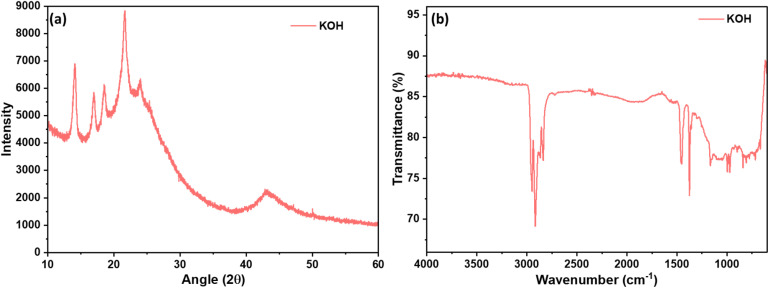
(a) XRD pattern and (b) FTIR of polymer-carbon composite.

The structural features and surface chemistry of the material were examined using X-ray diffraction (XRD) and Fourier-transform infrared spectroscopy (FTIR). The XRD patterns (Fig. 2a) show diffuse reflections indicative of largely amorphous carbon, with minor peaks around 13°, 17°, 19°, and 22° suggesting residual PE and PP domains. Broad humps near 23° and 43° correspond to the (002) and (100) planes of disordered carbon. The presence of these patterns reflecting the presence of both the polymers and AC.

FTIR spectra (Fig. 2b) indicate the presence of surface functional groups, with bands around 2850 cm^−1^ and 1400 cm^−1^ corresponding to C–H stretching and bending in PE/PP, and a peak around 1100 cm^−1^ characteristic of the AC.^47^ These observations are consistent with prior reports on pure and mixed PE/PP systems,^48,49^ validating the presence of residual polymer domains in the synthesized composites. The polymer peaks are more semicrystalline and showed sharp peaks in ∼2900 and ∼1400 regions, whereas the carbon peaks are blunt because of amorphous nature and showed peaks in the fingerprint region. C=O carbonyl groups of carbon is visible at ∼1700 region, because of surface oxidation of carbon as well as polymer, however, the intensity is relatively low. The corresponding peaks of both the polymers and carbon reflect the presence of the composite and is anticipated to have intermolecular interactions between these two entities.^50^

**Fig. 3:**
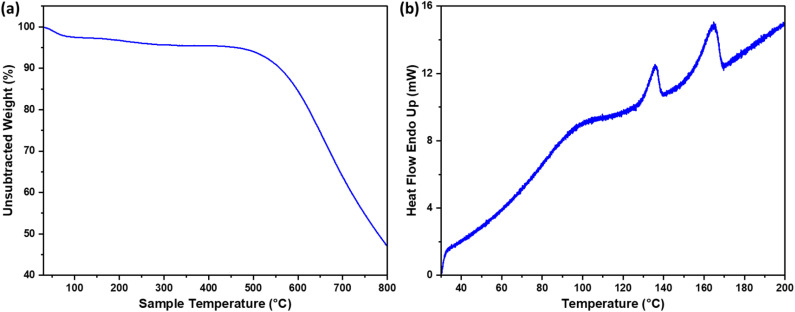
(a) TGA and (b) DSC curves of polymer-carbon composite made of MPW and palm fiber fronds.

The thermogravimetric analysis (TGA) curves (Fig. 3a) show the decomposition behavior of the polymer–carbon composite. A small mass loss of approximately 4% below 400 °C corresponds to the decomposition of the polymer fraction, confirming the presence of residual PE and PP in the composite. This also indicates that the chosen pyrolysis temperature of 550 °C effectively converts the biomass into carbon while retaining the majority of the carbon matrix. At higher temperatures, progressive decomposition of the composite is observed, and by 800 °C, over 50% of the carbon is degraded.

Differential scanning calorimetry (DSC, Fig. 3b) further confirms the presence of mixed plastics, with endothermic peaks corresponding to the melting points of PE (∼130 °C) and PP (∼165 °C). The spectra also reveal moisture loss at lower temperatures. The observed thermal behavior, together with physical intermolecular interactions between the polymer and carbon, contributes to the mechanical integrity of the composite.

**Fig. 4:**
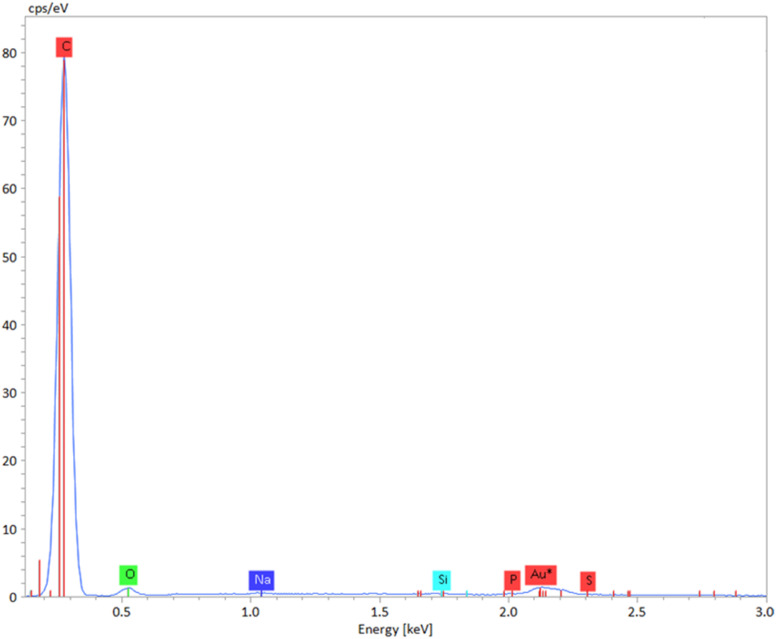
EDX of AC-polymer composite.

Moreover, EDX (Energy-Dispersive X-ray) analysis (Fig. 4) shows that the composite contains ∼96 wt% carbon and ∼4 wt% oxygen, indicating efficient conversion of biomass into carbon-rich char. The oxygen is attributed to surface oxygenated groups on the carbon and partial oxidation of the polymer, which promote physical binding and enhance intermolecular interactions within the composite.

### 3.3. Adsorption performance

**Fig. 5:**
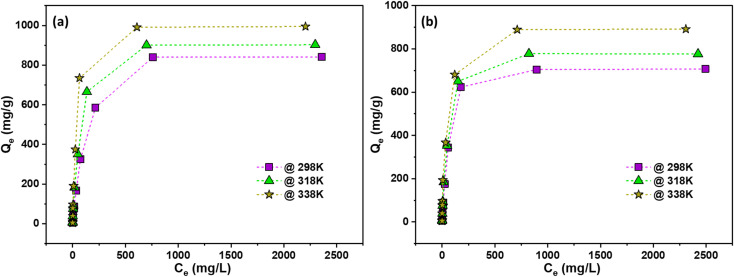
*C*_e_ *vs. Q*_e_ plot of adsorption of (a) RhB, and (b) NB dyes at different temperatures.

Fig. 5 presents the adsorption isotherms of the synthesized AC, showing the relationship between the equilibrium solution concentration (*C*_e_) and the adsorbed amount per unit mass (*Q*_e_). At 298 K, the AC exhibits a maximum adsorption capacity of 841 mg g^−1^ for RhB and 712 mg g^−1^ for NB. The adsorption performance improves with increasing temperature (318 K and 338 K), indicating enhanced dye uptake likely due to greater molecular mobility and improved accessibility of the AC pores. These results highlight the material's efficiency and temperature-dependent adsorption behavior.

### 3.4. Adsorption isotherm analysis

To understand the equilibrium behavior of dyes on the AC, experimental data were fitted to multiple isotherm models, including Langmuir,^43^ Freundlich,^51^ Temkin,^52,53^ Redlich–Peterson, as well as Toth and Dubinin–Radushkevich models. The Langmuir model assumes monolayer adsorption on uniform sites, while Freundlich describes multilayer adsorption on heterogeneous surfaces. Temkin accounts for adsorbent–adsorbate interactions with linearly decreasing heat of adsorption, and Redlich–Peterson combines features of Langmuir and Freundlich for heterogeneous surfaces.

To evaluate the suitability and predictive capability of the selected adsorption isotherm models for the current study, a systematic two-step approach was implemented. In the first step, the experimental adsorption data were directly compared with the values predicted by each isotherm model. This comparison enabled an initial assessment of how closely each model represents the observed adsorption behavior, providing insights into the nature of the adsorbent–adsorbate interactions, such as whether adsorption occurs as a monolayer or multilayer, on homogeneous or heterogeneous surfaces.

In the second step, each model was assessed using the correlation coefficient (*R*^2^). The *R*^2^ value reflects the degree of agreement between the experimental and predicted data, with higher values indicating a more accurate representation of the adsorption process. This two-pronged evaluation not only identifies the most appropriate isotherm model for describing the equilibrium behavior but also helps to interpret underlying adsorption mechanisms, including surface heterogeneity, energy distribution, and potential interactions between adsorbate molecules and active sites on the adsorbent surface.

**Fig. 6:**
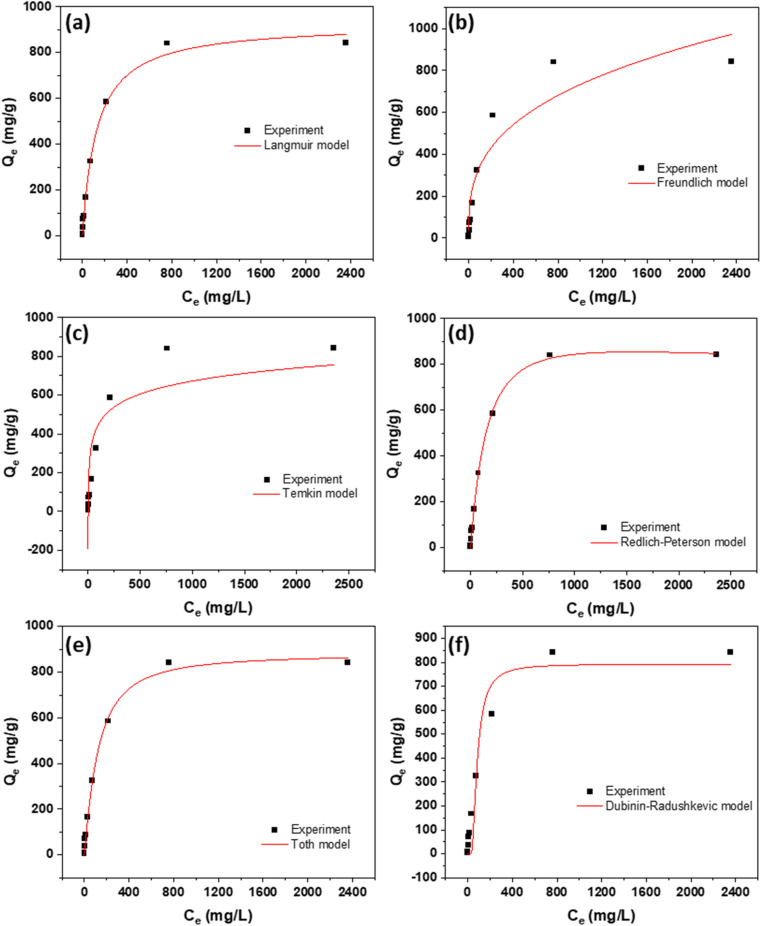
Isotherms using (a) Langmuir, (b) Freundlich, (c) Temkin, (d) Redlich–Peterson, (e) Toth, and (f) Dubinin–Radushkevic models for the adsorption of RhB dye at 298K.

**Fig. 7:**
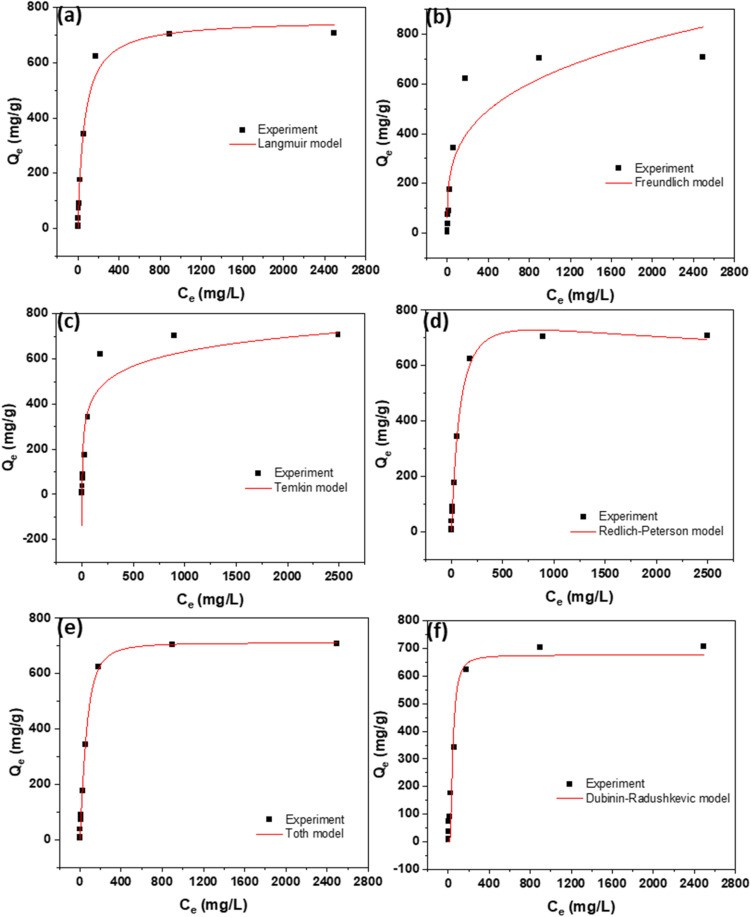
Isotherms using (a) Langmuir, (b) Freundlich, (c) Temkin, (d) Redlich–Peterson, (e) Toth, and (f) Dubinin–Radushkevic models for the adsorption of NB dye at 298K.

The results of isotherm analysis are presented in Fig. 6 and 7, illustrating the equilibrium adsorption behavior of the dyes on the AC. Table 2 and 3 complement these findings by listing the equations of all evaluated isotherm models, along with their corresponding parameters and values, allowing for a detailed comparison of adsorption characteristics. Among the models tested, the Langmuir isotherm provided the best fit to the experimental data, indicating that dye adsorption occurs predominantly as a monolayer on a surface with adsorption sites. This highlights the uniformity of the active sites on the AC surface and confirms the applicability of the Langmuir model for describing the adsorption mechanism in this system.

**Table 2:** Various isotherms at 298K in the removal of RhB dye

Model	Independent	Dependent	Equation	Parameters	Values
Langmuir nonlinear	*C*_e_	*Q*_e_	(*K*_L_ × *q*_m_ × *C*_e_)/(1 + (*K*_L_ × *C*_e_))	*q*_m_	927.0988
				*K*_L_	0.00776
				*R* ^2^	0.99486
Freundlich nonlinear	*C*_e_	*Q*_e_	*K*_F_ × *C*^(1/*n*)^_e_	*n*	3.02855
				*K*_F_	74.89236
				*R* ^2^	0.90816
Temkin	*C*_e_	*Q*_e_	((*R* × *T*)/*b*_T_) × (ln(*A*_T_ × *C*_e_))	*b*_T_	25.59002
				*A*_T_	1.04016
				*R* ^2^	0.81857
Redlich peterson	*C*_e_	*Q*_e_	(*K*_RP_ × *C*_e_)/(1 + (*a*_RP_ × *C*^bRP^_e_))	*K*_RP_	5.81443
				*a*_RP_	0.00288
				*b*_RP_	1.10361
				*R* ^2^	0.99796
Toth	*C*_e_	*Q*_e_	(*K*_t_ × *C*_e_)/(*a*_t_ + *C*_e_^*t*^)^(1/*t*)^	*K*_*t*	880.45842
				*a*_*t*	808.00836
				*T*	1.32475
				*R* ^2^	0.99651
Dubinin radushkevich	*C*_e_	*Q*_e_	*q*_DR_ × exp(−*B*_DR_ × (*R* × *T* × (ln(1 + (1/*C*_e_))))^2^)	*q*_DR_	793.02564
				*B*_DR_	8.56 × 10^−4^
				*E* = 1/sqrt(2 × *B*_DR_)	24.17249
				*R* ^2^	0.94069

**Table 3:** Various isotherms at 298K in the removal of NB dye

Model	Independent	Dependent	Equation	Parameters	Values
Langmuir nonlinear	*C*_e_	*Q*_e_	(*K*_L_ × *q*_m_ × *C*_e_)/(1 + (*K*_L_ × *C*_e_))	*q*_m_	756.27328
				*K*_L_	0.01572
				*R* ^2^	0.9911
Freundlich nonlinear	*C*_e_	*Q*_e_	*K*_F_ × *C*^(1/*n*)^_e_^)^	*n*	3.53345
				*K*_F_	90.65097
				*R* ^2^	0.85881
Temkin	*C*_e_	*Q*_e_	((*R* × *T*)/*b*_T_) × (ln(*A*_T_ × *C*_e_))	*b*_T_	26.99099
				*A*_T_	0.97801
				*R* ^2^	0.87698
Redlich peterson	*C*_e_	*Q*_e_	(*K*_RP_ × *C*_e_)/(1 + (*a*_RP_ × *C*^bRP^_e_))	*K*_RP_	9.2785
				*a*_RP_	0.00564
				*b*_RP_	1.10638
				*R* ^2^	0.99782
Toth	*C*_e_	*Q*_e_	(*K*_t_ × *C*_e_)/(*a*_t_ + *C*_e_^*t*^)^(1/*t*)^	*K*_*t*	711.44511
				*a*_*t*	4409.82296
				t	1.84495
				*R* ^2^	0.99856
Dubinin radushkevich	*C*_e_	*Q*_e_	*q*_DR_ × exp(−*B*_DR_ × (*R* × *T* × (ln(1 + (1/*C*_e_))))^2^)	*q*_DR_	676.00842
				*B*_DR_	2.49 × 10^−4^
				*E* = 1/sqrt(2 × *B*_DR_)	44.79757
				*R* ^2^	0.95111

### 3.5. Thermodynamic analysis

**Fig. 8:**
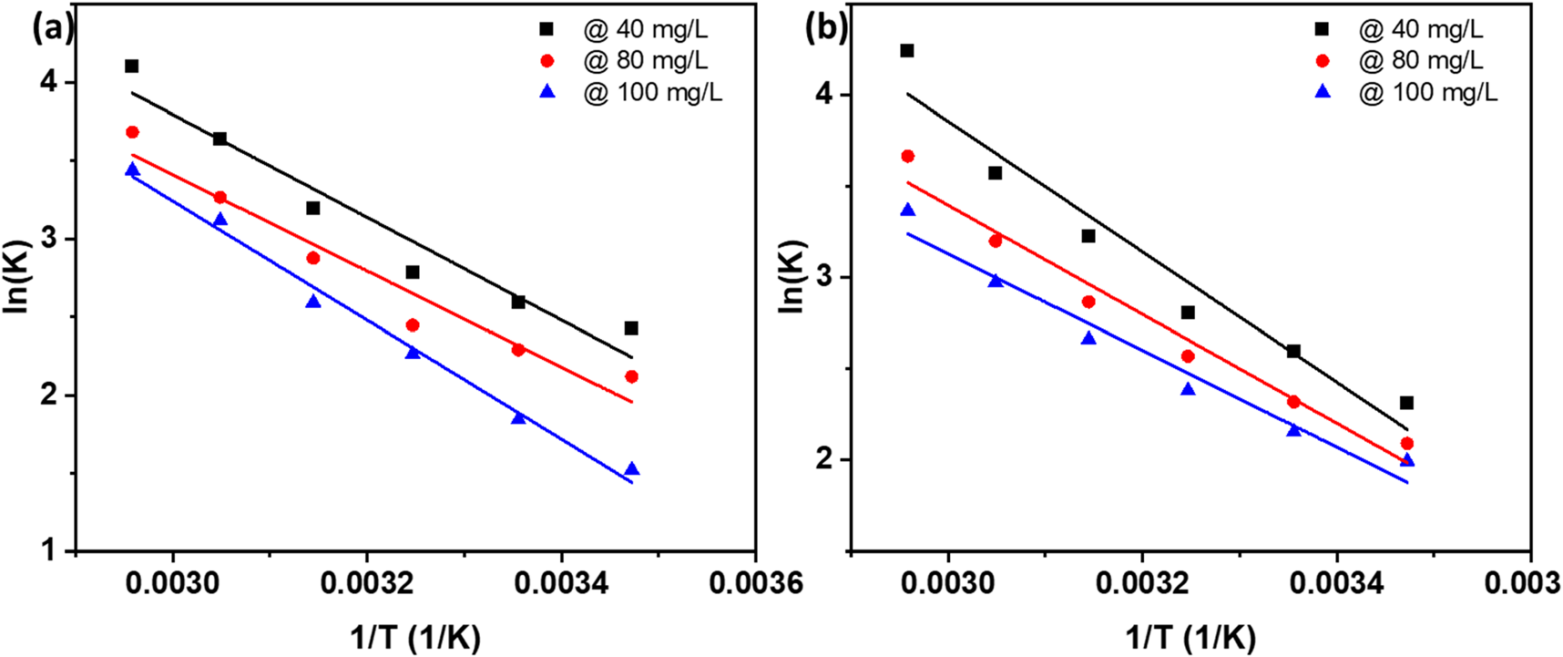
Thermodynamics of adsorption of (a) RhB and (b) NB dyes.

The thermodynamic parameters for the adsorption of RhB and NB—Δ*H*°, Δ*S*°, and Δ*G*°—are shown in Fig. 8 and summarized in Table 4 and 5. Positive Δ*H*° values indicate that the adsorption process is endothermic, as evidenced by the increased adsorption capacity with rising temperatures from 288 K to 338 K. The positive Δ*S*° values reflect the strong affinity of the AC for the dyes, suggesting increased disorder at the solid–solution interface and greater mobility of dye molecules on the AC surface, which enhances adsorption efficiency.^43^

**Table 4:** Thermodynamic values using AC for RhB adsorption

(K)	Δ*G*			Δ*H*			Δ*S*		
	40 mg L^−1^	80 mg L^−1^	120 mg L^−1^	40 mg L^−1^	80 mg L^−1^	120 mg L^−1^	40 mg L^−1^	80 mg L^−1^	120 mg L^−1^
288	−5803	−5071.35	−3643.05	27 344.59	25 609.58	31 676.49	113.5786	105.1713	121.9705
298	−6417.57	−5671.56	−4564.16						
308	−7131.61	−6262.7	−5791.72						
318	−8434.51	−7602.6	−6846.5						
328	−9926.18	−8902.23	−8497.26						
338	−11525.6	−10348.6	−9660.92						

**Table 5:** Thermodynamic values using AC for NB adsorption

(K)	Δ*G*			Δ*H*			Δ*S*		
	40 mg L^−1^	80 mg L^−1^	120 mg L^−1^	40 mg L^−1^	80 mg L^−1^	120 mg L^−1^	40 mg L^−1^	80 mg L^−1^	120 mg L^−1^
288	−5539.84	−5006.14	−4774.49	29 751.41	24 865.93	22 050.68	121.3285	102.8446	92.18346
298	−6435.21	−5745.77	−5335.57						
308	−7184.95	−6581.19	−6097.64						
318	−8533.63	−7581.05	−7039.49						
328	−9739.57	−8728.93	−8110.27						
338	−11918.7	−10307.1	−9462.55						

Negative Δ*G*° values confirm the spontaneity and feasibility of the adsorption process, demonstrating that dye uptake occurs readily without external energy input.^43,47^ This negative Gibbs free energy change points to a high preference of the dye molecules for the AC, corroborating the spontaneous adsorption mechanism. This spontaneous nature is essential for practical applications, ensuring that the adsorption process occurs readily without requiring external energy input. These observations are consistent with previous studies reporting spontaneous and endothermic adsorption on biomass-derived adsorbents, including Rhodamine 6G and Indigo Carmine adsorption on coconut shell,^31^ arsenic on coconut husk,^54^ methylene blue (MB) onto corncorb,^55^ anionic and cationic dyes onto rubber seed shell and rubber seed,^47^ and MB onto oil palm fiber.^43^

### 3.6. Factors affecting adsorption

**Fig. 9:**
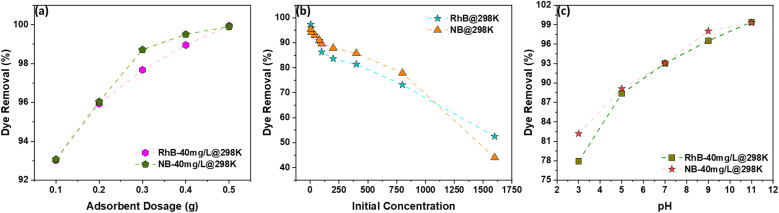
(a) Adsorbent dosage *vs.* % dye removal, (b) initial concentration *vs.* % dye removal, and (c) (b) pH *vs.* % dye removal.

The efficiency of dye removal by AC is strongly influenced by adsorbent dosage, initial dye concentration, solution pH, and temperature. Increasing the AC dosage from 0.1 g to 0.5 g improved dye removal from 93.7% to nearly 100%, as more active sites became available for adsorption (Fig. 9a).^47^ Beyond this threshold, additional AC did not further enhance removal, indicating sufficient sites for complete uptake and allowing for optimization to reduce excess adsorbent use and waste.

The initial dye concentration also affects adsorption performance (Fig. 9b). At low concentrations (≤40 mg L^−1^), the AC efficiently utilizes its active sites, while higher concentrations lead to site saturation, reducing removal efficiency. This demonstrates that the AC has a finite number of active sites that determine its maximum uptake capacity.

Solution pH plays a pivotal role by affecting the surface charge of AC and the ionization state of the dyes (Fig. 9c). Under acidic conditions, high H^+^ concentrations compete with cationic dyes for adsorption sites, reducing uptake. At higher pH, the AC surface becomes negatively charged due to alkaline activation, enhancing electrostatic attraction with positively charged dye molecules. In particular, KOH-activated AC introduces alkaline functional groups that favor interaction with cationic dyes, resulting in improved adsorption efficiency.^47^

**Fig. 10:**
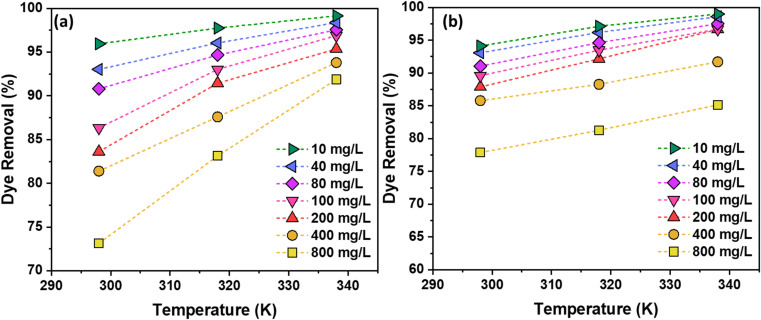
Temperature *vs.* % dye removal for (a) RhB, and (b) NB dyes.

Temperature further influences adsorption (Fig. 10). Increasing the solution temperature enhances dye molecule mobility, facilitating diffusion to the AC surface and promoting interactions with active sites, which results in higher removal efficiencies. Collectively, these results demonstrate that optimal adsorption is achieved by carefully balancing adsorbent dosage, dye concentration, solution pH, and temperature, ensuring maximum efficiency while minimizing material use.

### 3.7. Modeling of sorption kinetics

**Fig. 11:**
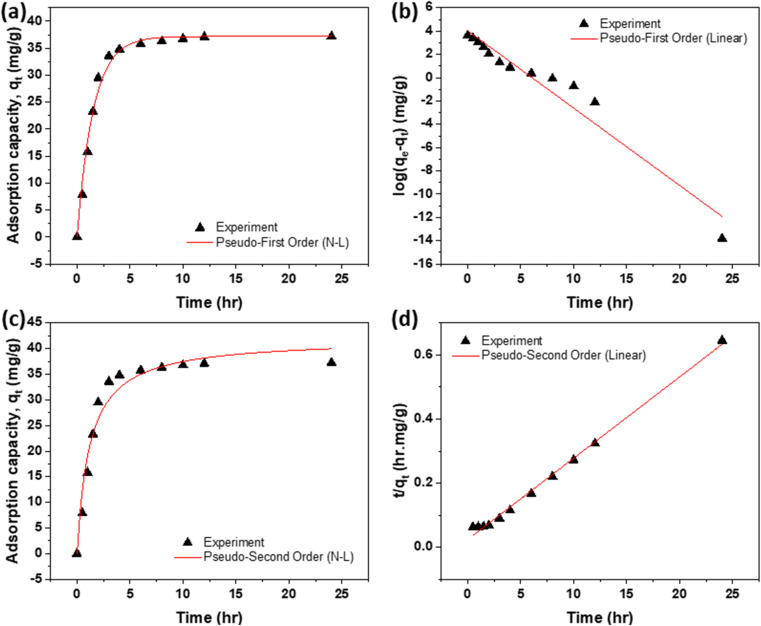
Saturation uptake of RhB dye: (a) and (b) non-linear and linear forms of PFO; and (c) and (d) non-linear and linear forms of PSO.

**Fig. 12:**
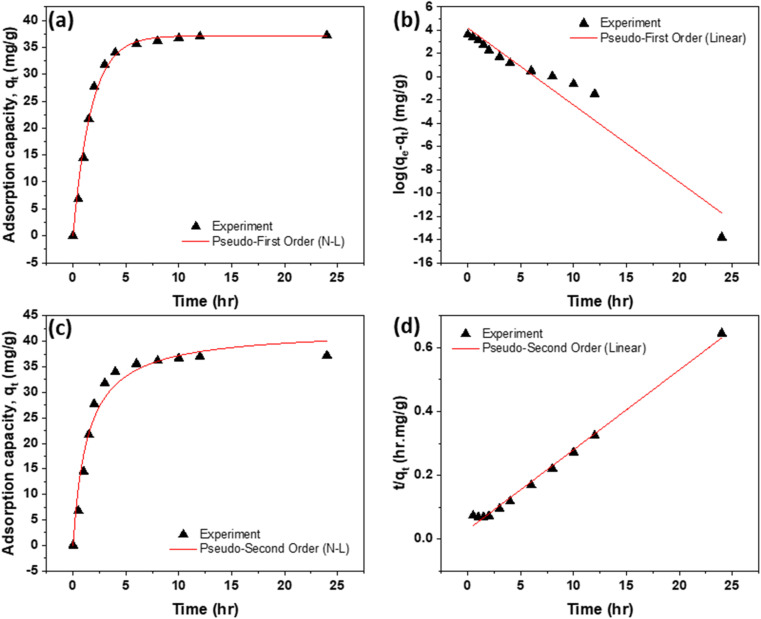
Saturation uptake of NB dye: (a) and (b) non-linear and linear forms of PFO; and (c) and (d) non-linear and linear forms of PSO.

The adsorption kinetics of dyes at 298 K and an initial concentration of 40 mg L^−1^ were evaluated using pseudo-first-order (PFO) and pseudo-second-order (PSO) models (Fig. 11 and 12). Kinetic plots were employed to determine the rate constants and equilibrium sorption capacities (*q*_e_), as summarized in Table 6 and 7. Both models aligned well with the experimental data; however, the PSO model—originally proposed by one of the authors^56^ and now widely used—provided a superior fit during the initial adsorption phase, indicating that chemisorption governed the rapid uptake. Conversely, the PFO model better described the later stage, suggesting diffusion-controlled transport as equilibrium was approached.

**Table 6:** Parameters and their values used in Kinetic study for RhB adsorption

Model	Independent	Dependent	Equation	Parameters	Values
PFO (non-linear)	Time	log(*q*_e_−*q*_t_)	ln(*q*_e_) − (*k*_1_ × *t*)/2.303	*q*_e_	56.21
				*k*_1_	1.53
				*R* ^2^	0.94
PFO (linear)	Time	Adsorption capacity, *q*_t_	*q*_e_ × (1−exp(−(*k*/2.303) × *t*))	*q*_e_	37.17
				*k*	1.51
				*R* ^2^	0.99
PSO (non-linear)	Time	*t*/*q*_t_	(1/(*k*_2_ × *q*_e_^2^)) + (*t*/*q*_e_)	*q*_e_	39.40
				*k*_2_	0.03
				*R* ^2^	1.00
PSO (linear)	Time	Adsorption capacity, *q*_t_	(*k* × *q*_e_^2^ × t)/(1 + *k* × *q*_e_ × *t*)	*q*_e_	41.95
				*k*	0.02
				*R* ^2^	0.96

**Table 7:** Parameters and their values used in Kinetic study for NB adsorption

Model	Independent	Dependent	Equation	Parameters	Values
PFO (non-linear)	Time	log(*q*_e_−*q*_t_)	ln(*q*_e_)−(*k*_1_ × *t*)/2.303	*q*_e_	66.20
				*k*_1_	1.53
				*R* ^2^	0.94
PFO (linear)	Time	Adsorption capacity, *q*_t_	*q*_e_ × (1−exp(−(*k*/2.303) × *t*))	*q*_e_	37.15
				*k*	1.34
				*R* ^2^	0.99
PSO (non-linear)	Time	*t*/*q*_t_	(1/(*k*_2_ × *q*_e_^2^)) + (*t*/*q*_e_)	*q*_e_	39.92
				*k*_2_	0.02
				*R* ^2^	0.99
PSO (linear)	Time	Adsorption capacity, *q*_t_	(*k* × *q*_e_^2^ × t)/(1 + *k* × *q*_e_ × *t*)	*q*_e_	42.37
				*k*	0.02
				*R* ^2^	0.97

Saturation of adsorption sites occurred within 6 h, attributed to the interconnected pores and inner microcavities created by polymer incorporation and heat treatment. These structural features enhanced active site accessibility and promoted rapid dye transport. Additionally, thermal treatment improved surface hydrophilicity, facilitating water penetration and interaction between dye molecules and the polymer–carbon matrix. Overall, the combined effect of heat treatment and polymer addition accelerated adsorption kinetics, yielding high dye removal efficiency.

## 4. Conclusion

This study presents a sustainable method for producing activated carbon (AC) from waste polyolefin and palm fronds *via* a dissolution-assisted co-processing route. The resulting AC exhibited a highly porous structure with abundant surface functional groups, enabling efficient adsorption of cationic dyes, with maximum capacities of 841 mg g^−1^ and 707 mg g^−1^ for RhB and NB, respectively. Adsorption behavior was best described by the Langmuir model, confirming monolayer coverage on homogeneous adsorption sites. Kinetic studies revealed a rapid initial uptake followed by gradual saturation of adsorption sites, reaching equilibrium within 6 h, while thermodynamic analysis indicated that the process is spontaneous and endothermic.

The incorporation of polymer during AC synthesis and subsequent heat treatment significantly enhanced the structural and functional properties of the composites. These modifications promoted interconnected porosity, improved accessibility of active sites, and increased surface hydrophilicity, collectively accelerating adsorption kinetics and improving overall dye removal efficiency. Systematic evaluation of operational parameters—including adsorbent dosage, dye concentration, solution pH, and temperature—provided insights for optimizing adsorption performance.

Importantly, the dissolution-assisted casting approach enables the preparation of mold-shaped AC composites that can be produced in practical geometries such as cylindrical bodies and subsequently cut into flakes, cubes, or other desired forms. This morphology offers clear advantages for industrial handling and scale-up, including improved processability, easier solid–liquid separation, and better compatibility with fixed-bed or continuous adsorption systems. Notably, despite the shaped form, the adsorption capacity achieved with this morphology remains comparable to that of conventional powdered AC, demonstrating its potential as a scalable and application-ready adsorbent for wastewater treatment.

## Author contributions

Junaid Saleem: conceptualization, investigation, formal analysis, supervisor, writing – review & editing. Zubair Khalid Baig Moghal: formal analysis, investigation, validation, writing – review & editing. Gordon McKay: validation, formal analysis.

## Conflicts of interest

There are no conflicts to declare.

## Data Availability

The original contributions presented in the study are included in the article, further inquiries can be directed to the corresponding author.
